# REPLY TO THE AUTHORS: Re: Endourologic strategies for a minimally invasive management of urinary tract stones in patients with urinary diversion

**DOI:** 10.1590/S1677-5538.IBJU.2018.0123.1

**Published:** 2018

**Authors:** FangLing Zhong, Gurioli Alberto, GuangMing Chen, Wei Zhu, FuCai Tang, Guohua Zeng, Ming Lei

**Affiliations:** 1 Department of Urology, Minimally Invasive Surgery Center, the First Affiliated Hospital of Guangzhou Medical University, Guangdong Key Laboratory of Urology, Guangzhou, China; 2 Department of Urology, Turin University of Studies, Turin, Italy

*To the editor,*

We appreciated the valuable comments on our recent published article in the International Braz J Urol (1, 2). We agree with the commentary, to our knowledge, these studies have shown that the complicated patients with lower tract stone were treated by endoscopic management.

In patients with reservoir stone after urinary diversion, stone management present unique challenges. In our research, 3 patients with reservoir stones following urinary diversion were treated by transurethral neo-bladder lithotripsy, and one patient had 6 mm residual stone postoperatively and received subsequently conservative watching treatment (2). Recently, percutaneous pouch access and laparoscopic techniques to facilitate the treatment of lower tract stones has become popular (3,4).

With the advancement of equipments and increasing experience, the surgical management of urolithiasis in patients with urinary diversion are varied, individualized consideration and comprehensive evaluation must be taken into account, which depending upon diversion type, patient fitness, stone size, stone location, available resource and surgeon experience (5,6).
